# Norm-based measures of inequality: A property-focused evaluation

**DOI:** 10.1371/journal.pone.0337916

**Published:** 2026-04-21

**Authors:** Muhammad Hamza, Beomsu Baek, Joongyang Park, Youngsoon Kim

**Affiliations:** 1 Department of Bio and Medical Big Data, Gyeongsang National University, Jinju-si, Gyeongsangnam-do, Republic of Korea; 2 Department of Information and Statistics, Gyeongsang National University, Jinju-si, Gyeongsangnam-do, Republic of Korea; Instituto Superior de Contabilidade e Administracao de Lisboa - Instituto Politecnico de Lisboa (ISCAL-IPL), PORTUGAL

## Abstract

Norm-based inequality measures are developed within the unequally distributed/relative unequally distributed (UD/RUD) framework by applying the *L*_1_ norm and the squared *L*_2_ norm to the cumulative distribution and quantile function (CDQF), the quantile function (QF), and their combined representation. All six indices maintain key properties such as scale, replication, and translation invariance, as well as anonymity, and their behavior under Pigou-Dalton transfers and subgroup decomposition is examined analytically. The indices based on the cumulative distribution and quantile function combine the vertical and horizontal gaps, giving them representation decomposability and making them the most informative overall measures of inequality. A Monte Carlo study on six contrasting income distributions corroborates these theoretical advantages, confirming finite, stable values even for mixed-sign and heavy-tailed supports.

## Introduction

Measurement of inequality is a key topic in economics. Many studies have developed inequality indexes. Hao and Naiman [1] and Cowell [2] present comprehensive lists of inequality indexes. We call the indexes in the lists the conventional indexes. Many studies also considered the desirable properties of inequality indexes. [3–11,12,13] The commonly mentioned properties are scale invariance, replication invariance, anonymity, transfer principle, and normalization. Scale invariance means that the value of an inequality index does not change when all incomes are multiplied by the same positive constant. Replication invariance means that the index remains unchanged when the population is replicated.

Anonymity means that the index depends only on the income distribution, not on the identity of the individuals. The transfer principle means that a transfer from a richer person to a poorer person should reduce inequality, provided that the income ranking does not change. These properties serve two purposes in research. They are validation and development. We can validate if an inequality index satisfies these properties to determine if it is robust, reliable, and meaningful. If an index fails to meet these properties, it may not accurately measure the inequality. We can develop new inequality indexes to satisfy these properties. Then, we ensure that new indexes are reliable and accurately measure inequality. The new indexes can address the limitations of conventional indexes and offer improved assessments of inequality. For example, Atkinson [3] and Sen [4] showed that the summary inequality indexes did not satisfy the desirable properties and proposed new inequality indexes meeting the desirable properties. Allison [5] evaluated the Gini coefficient, the coefficient of variation, and the Theil index concerning the desirable properties. Shorrocks [6] proposed a decomposable index for income mobility and inequality satisfying the desirable properties. Foster [7] showed that the Theil index is the only index satisfying all the desirable properties. Ebert [8] introduced a set of inequality measures meeting the desirable properties. Litchfield [9] provided a comprehensive overview of the conventional indexes with respect to the desirable properties. Jenkins et al. [10] considered the choice of inequality indexes based on the desirable properties. Clementi et al. [11] proposed the Zanardi index [14,15] as an appropriate measure of inequality and showed that it satisfies the desirable properties. Chakravarty et al. [12] reviewed the desirable properties and applied them to multidimensional indexes. Billette de Villemeur and Leroux [13] translated the desirable properties of the weight function and introduced criterion-ranking inequality indexes.

We can consider inequality for many variables such as income, wealth, opportunity, and health. This paper uses income as a representative variable. Recently, Park et al. [16,17] and Kim et al. [18] argued that conventional indexes are based on a different conceptualization of inequality and proposed a new approach to measuring inequality. The proposed approach is based on unequally distributed (UD) income or relative UD (RUD) income rather than income itself, and it evaluates the degree of inequality using the *L*_1_ and *L*_2_ norms. Here, a norm is used as a measure of the distance between the observed distribution and perfect equality. Although earlier studies introduced the UD and RUD income-based approaches and the associated norm-based measures, their theoretical properties have not yet been examined in a sufficiently unified way. In particular, the role of the squared *L*_2_ norm and the combined use of the cumulative distribution function, quantile function, and cumulative-distribution-and-quantile-function representation deserve clearer emphasis. This paper contributes to the literature by providing a systematic property-based evaluation of these norm-based measures and by clarifying how they differ from conventional inequality indexes. We organize this paper as follows. The next section reviews the desirable properties. The following section summarizes the UD and RUD income-based approaches and their norm indexes and proposes the squared *L*_2_ norm instead of the *L*_2_ norm. The subsequent section investigates the properties of the norm indexes. The paper ends with a brief conclusion.

## Desirable properties of inequality indexes

This section briefly reviews the desirable properties commonly used to evaluate inequality indexes. These properties provide the basis for assessing the norm-based indexes introduced later in the paper. Suppose a population consisting of *n* individuals. The income distribution of the population is denoted by $𝐲=\left(y_{1},y_{2},\cdots ,y_{n}\right)$, where *y*_*i*_ is the income of the *i*-th individual. Without loss of generality, we can assume that the incomes are ordered, i.e., $y_{1}\leq y_{2}\leq \ldots \leq y_{n}$. The total income and mean income are denoted by $S_{y}=\sum_{i=1}^{n}y_{i}$ and $\mu_{y}=S_{y}/n$, respectively. Statistically, the usual representations of income distribution are the cumulative distribution function (CDF), the quantile function (QF), and the Lorenz curve. If the CDFs, QFs, or Lorenz curves of **y**_1_ and **y**_2_ are the same, we say that **y**_1_ and **y**_2_ have the same distribution. Equality, also referred to as perfect equality, is denoted by ${𝐲}_{pe}=\left(\mu_{y},\mu_{y},\cdots ,\mu_{y}\right)$.

Litchfield [9] defines inequality as the dispersion of a distribution. McKay [19] defines inequality as variations in living standards across a whole population. Chakravarty [20] mentions that income inequality represents interpersonal income differences within a given population. Cowell [2, p. 7] defined an income inequality index as a numerical representation of the interpersonal differences in income within a given population. According to these definitions, conventional inequality indexes measure variation in the income values *y*_*i*_. All conventional indexes except the Gini coefficient measure variation in the values *y*_*i*_ around $\mu_{y}$. The Gini coefficient measures the distance between Lorenz curves for **y** and **y**_*pe*_. We denote by I(𝐲) an income inequality index that measures variation in the values *y*_*i*_.

As mentioned in the previous section, the desirable properties of inequality indexes are scale invariance, replication invariance, anonymity, the transfer principle, normalization, and decomposability. This section reviews these properties for I(𝐲). Scale invariance, introduced by Allison [5], ensures that an income inequality index remains consistent regardless of the scaling of income. An index I(𝐲) is scale-invariant if I(𝐲)=I(α𝐲) for any α>0. This property is crucial for comparing inequality across countries or regions with different income levels and monetary units.

Replication invariance means that duplicating individuals in a population does not change the inequality measure. Replication invariance is essential when studies compare inequality across different populations. An index I(𝐲) is replication-invariant if duplicating the population with the same income distribution does not change the value of the index, i.e., $I\left(𝐲\right)=I\left({𝐲}_{r}\right)$ where ${𝐲}_{r}=\left(y_{1},y_{1},y_{2},y_{2},\ldots ,y_{n},y_{n}\right)$.

Anonymity ensures that an inequality index is unaffected by permutations of the incomes. An index I(𝐲) satisfies anonymity if it is unchanged by the permutation of incomes among individuals. Anonymity is a fundamental principle in inequality measurement. It means that an inequality index depends only on the income distribution.

Pigou [21] introduced the transfer principle. Later Dalton [22] recognized it. Therefore, it is also known as the Pigou-Dalton principle. It states that a transfer of income from a richer individual to a poorer individual reduces inequality. Let *y*_*i*_ < *y*_*j*_ and ϵ be a small positive amount such that $\left(y_{i}+\epsilon \right)\leq \left(y_{j}-\epsilon \right)$. An index I(𝐲) satisfies the transfer principle if $I\left({𝐲}_{tr}\right)\leq I\left(𝐲\right)$, where ${𝐲}_{tr}=\left(y_{1},y_{2},\ldots ,y_{i}+\epsilon ,\ldots ,y_{j}-\epsilon ,\ldots ,y_{n}\right)$.

Normalization ensures that an inequality index has upper and lower bounds, typically with 0 representing perfect equality and 1 representing maximum inequality. An index I(𝐲) is normalized if it has upper and lower bounds. Normalization makes it easy to interpret and compare inequality across different studies. The Gini coefficient is one of the normalized inequality indexes. Chen et al. [23] and Raffinetti et al. [24] further contributed to normalization in inequality measurement. They addressed normalization issues when dealing with negative incomes.

Decomposability usually refers to subgroup decomposability. Subgroup decomposability, introduced by Bourguignon [25] and further developed by Shorrocks [6], means that total inequality should be related to inequality within subgroups and between subgroups. An index I(𝐲) is decomposable if I(𝐲)=W(𝐲)+B(𝐲) where *W* represents within-group inequality and *B* represents between-group inequality. Subgroups are usually formed by gender, age, or ethnicity. It allows policymakers to identify which subgroups contribute most to inequality and target subgroups for interventions.

## Norm inequality indexes of the UD and RUD income-based approaches

This section introduces the UD and RUD income-based approaches and presents the norm-based inequality indexes studied in this paper. It also introduces the squared *L*_2_-norm indexes used in the later analysis. Park et al. [16] introduced the UD income-based approach, and Park et al. [17] and Kim et al. [18] later developed it further. They argue that the values *y*_*i*_ contain information about both equality and inequality, and that the inequality component should be extracted from them.

Since $y_{i}=y_{1}+\left(y_{i}-y_{1}\right)$ for all *i*, we see that *n y*_1_ is equally distributed to *n* individuals, while $\left(S_{y}-ny_{1}\right)$ is unequally distributed to *n* individuals. We define $x_{i}=\left(y_{i}-y_{1}\right)$, i=1,2,…,n, as the UD incomes. Then $S_{x}=\left(S_{y}-ny_{1}\right)$ and $\mu_{x}=S_{x}/n$ denote the total and mean of the UD incomes and $𝐱=\left(x_{1},x_{2},\ldots ,x_{n}\right)$ represents how the total UD income *S*_*x*_ is unequally distributed to *n* individuals.

A feature of the UD income-based approach is that it employs a different definition of inequality from the one given in the previous section. Cowell [2, p. 1] defines inequality as a departure from equality. Hao and Naiman [1] also define inequality in terms of equality as the absence of equality. These definitions require equality. We cannot define or measure inequality without a notion of equality. Zero variation in **y** implies that **y** represents equality. Non-zero variation in **y** implies that **y** does not represent equality. However, that does not mean that the variation of **y** is the departure of **y** from equality. We denote the UD income distribution for ${𝐲}_{pe}$ by ${𝐱}_{pe}=\left(0,0,\ldots ,0\right)$. The UD income-based approach measures inequality by the departure of **x** from **x**_*pe*_, not the variation within **y** or **x**. The conventional indexes consider inequality a property of an income distribution **y**. The UD and RUD income-based approaches consider inequality a relationship between two UD income distributions, **x** and **x**_*pe*_. The UD and RUD income-based approaches produce indexes that differ substantially from conventional indexes.

We can derive **x**, *S*_*y*_, and **x**_*pe*_ from **y** and ${𝐲}_{pe}$. Conversely, we can restore **y** and ${𝐲}_{pe}$ from **x**, *S*_*y*_, and **x**_*pe*_. The UD income approach does not involve any loss of information. Park et al. [17] consider three representations of the UD income distribution—the cumulative distribution function (CDF), the quantile function (QF), and the Lorenz curve—and two distance measures, namely the *L*_1_ norm and the *L*_2_ norm. Specifically, *L*_1_ and *L*_2_ norm distances between CDFs (QFs, Lorenz curves) of **x** and **x**_*pe*_ are obtained, normalized by $\mu_{y}$, and then proposed as inequality indexes. Kim et al. [18] showed that this is equivalent to *L*_1_ and *L*_2_ norm distances between CDFs (QFs, Lorenz curves) of **z** and **z**_*pe*_, where $z_{i}=x_{i}/\mu_{y}$s are the RUD incomes, $𝐳=\left(z_{1},z_{2},\ldots ,z_{n}\right)$, and ${𝐳}_{pe}=\left(0,0,\ldots ,0\right)$. The cumulative distribution function (CDF) and the quantile function (QF) convey different information about the income distribution of a finite population. The Lorenz curve and the quantile function convey equivalent information because the Lorenz curve is an integral of the quantile function. The departure of CDF of **z** from CDF of **z**_*pe*_ is called the vertical departure, while the departure of QF of **z** from QF of **z**_*pe*_ is called the horizontal departure. Kim et al. [18] introduced the cumulative distribution and quantile function (CDQF) of **z** to integrate the vertical and horizontal departures by combining the CDF and the QF. They measured inequality using the *L*_1_ and *L*_2_ norms of the departure of the CDQF from perfect equality.

We can use variance instead of standard deviation as a dispersion measure. Similarly, we can use the square of the *L*_2_ norm instead of the *L*_2_ norm as a distance measure. We propose the squared *L*_2_-norm indexes, which are easily derived from the *L*_2_-norm indexes of Park et al. [17] and Kim et al. [18]. The squared *L*_2_ norm is not introduced only for computational convenience. It also has a useful interpretive role because it gives greater weight to larger departures from perfect equality and therefore makes the measure more sensitive to pronounced inequality. In addition, it is directly comparable to the relation between the standard deviation and the variance: both are based on the same underlying distance, but the squared form is often more convenient for theoretical analysis and decomposition. Table 1 shows the *L*_1_ and square of the *L*_2_ norm indexes for four representations of **z**. Here, $\mu_{z}$, *CV*_*z*_, *G*_*z*_, and *z*_*n*_ are the mean, coefficient of variation, Gini coefficient, and maximum of RUD incomes respectively. The subscripts *c*, *q*, *cq*, *l* denote the corresponding representations: cumulative distribution function (CDF), quantile function (QF), cumulative distribution and quantile function (CDQF), and Lorenz curve.

**Table 1:** RUD income-based inequality indexes.

	Distance measure	
Distribution representation	*L*_1_ norm	Square of *L*_2_ norm ($L_{2}^{2}\;$)
CDF	$L_{1,c}=\mu_{z}$	$L_{2,c}^{2}=\mu_{z}\left(1-G_{z}\right)$
QF	$L_{1,q}=\mu_{z}$	$L_{2,q}^{2}=\mu_{z}^{2}\left(1+CV_{z}^{2}\right)$
CDQF	$L_{1,cq}=L_{1,c}+L_{1,q}$	$L_{2,cq}^{2}=L_{2,c}^{2}+L_{2,q}^{2}$
Lorenz curve	$L_{1,l}=\frac{1}{2}\mu_{z}\left(1-G_{z}\right)$	not available

Negative income values are collected in practice. As mentioned in the previous section, Chen et al. [23] and Raffinetti et al. [24] considered normalization issues when dealing with negative income values. Negative income values indicate that income is not a ratio but an interval scale. The conventional indexes do not apply to variables of interval scale. [5] Beckman [26] studied the measurement of inequality for interval data. The UD and RUD income-based approaches do not suffer from negative and interval scale values. That is because the UD incomes are non-negative, and the difference between two interval values has a ratio scale. Therefore, the UD and RUD income-based approaches apply to interval scale variables.

## Properties of the norm inequality indexes

This section examines whether the proposed norm-based indexes satisfy the desirable properties reviewed in the previous section. The goal is to evaluate their theoretical behavior in a systematic way. Recently, Park et al. [16,17] and Kim et al. [18] introduced the UD and RUD income-based approaches to measuring inequality and proposed norm indexes based on a different conceptualization of inequality. The UD and RUD income-based approaches result in the same norm indexes. This section examines the norm indexes in Table 1 concerning the desirable properties. We denote a norm index in Table 1 by D(𝐳).

### Scale invariance

Assume ${𝐲}_{s}=\alpha 𝐲$ for any α>0. Then the minimum, sum and mean of **y**_*s*_ are $\alpha y_{1}$, $\alpha S_{y}$, and $\alpha \mu_{y}$. Therefore, **z**_*s*_ = **z**, $D\left({𝐳}_{s}\right)=D\left(𝐳\right)$, and D(𝐳) is scale-invariant.

### Replication invariance

Assume ${𝐲}_{r}=\left(y_{1},y_{1},y_{2},y_{2},\ldots ,y_{n},y_{n}\right)$. Then the minimum and mean of **y**_*r*_ are *y*_1_ and $\mu_{y}$. Therefore, ${𝐳}_{r}=\left(z_{1},z_{1},z_{2},z_{2},\ldots ,z_{n},z_{n}\right)$. **z**_*r*_ and **z** have the same CDF, QF, CDQF, and Lorenz curve. Consequently, $D\left({𝐳}_{r}\right)=D\left(𝐳\right)$ and D(𝐳) is replication-invariant.

### Anonymity

The UD and RUD income-based approaches assume $y_{1}\leq y_{2}\leq \ldots \leq y_{n}$. Therefore, the permutation of incomes among *n* individuals does not change **y** and **z**. D(𝐳) satisfies anonymity.

### Transfer principle

This subsection requires special attention because, under the UD/RUD framework, inequality is measured by departure from perfect equality rather than by variation alone. For this reason, the effect of a progressive transfer is not always the same as in the conventional framework.

Let *y*_*i*_ < *y*_*j*_ and ϵ be a small positive amount such that $\left(y_{i}+\epsilon \right)\leq \left(y_{j}-\epsilon \right)$. Such transfer of ϵ from *j*-th individual to *i*-th individual is called the progressive transfer preserving the mean. Let ${𝐲}_{tr}=\left(y_{1},y_{2},\ldots ,y_{i}+\epsilon ,\ldots ,y_{j}-\epsilon ,\ldots ,y_{n}\right)$. An index I(𝐲) is said to satisfy the transfer principle if $I\left({𝐲}_{tr}\right)\leq I\left(𝐲\right)$. As Ebert [8] mentioned, the transfer principle is about the variation of **y**. The progressive transfer decreases the variation of **y** and consequently decreases an inequality index measuring the variation of **y**. However, the UD and RUD income-based indexes measure the departure of **z** from **z**_*pe*_, not the variation of **z**. Table 1 shows that the variation of **z** is just a component of the departure of **z** from **z**_*pe*_. Therefore, we need to investigate the effect of the progressive transfer on inequality. In this paper, it is useful to distinguish between two different interpretations of transfer. A variation-reducing transfer lowers the dispersion of the income distribution in the conventional sense, whereas an equality-distance-reducing transfer brings the UD/RUD distribution closer to the benchmark of perfect equality. These two effects may coincide, but they need not be the same. A norm index D(𝐳) satisfies the transfer principle if $D\left({𝐳}_{tr}\right)\leq D\left(𝐳\right)$. This result should be interpreted carefully. It does not imply that redistribution is undesirable or that progressive transfers should be rejected as a policy principle. Rather, it shows that the usual transfer principle is tied to inequality measures based on dispersion, whereas the UD/RUD framework evaluates inequality as departure from perfect equality. Under this alternative interpretation, a progressive transfer may reduce dispersion while increasing the distance from the equality benchmark. The difference is therefore conceptual and metric-based, not a rejection of redistribution itself.

Since **z** and **z**_*tr*_ are different, unlike the invariance properties, the effect of a progressive transfer depends on how the departure of **z** from **z**_*pe*_ is evaluated. We can classify the indexes in Table 1 into two groups. One is the indexes involving only $\mu_{z}$, which are *L*_1,*c*_, *L*_1,*q*_ and *L*_1, *cq*_. The other is the indexes involving $\mu_{z}$ and a dispersion measure, *CV*_*z*_ or *G*_*z*_, which are *L*_*l*,*l*_, $L_{2,c}^{2}$, $L_{2,q}^{2}$, and $L_{2,cq}^{2}$. Noting $\mu_{z}=\left(\mu_{y}-y_{1}\right)/\mu_{y}$, we see that the progressive transfer preserving the mean decreases *L*_1,*c*_, *L*_1,*q*_ and *L*_1, *cq*_ only when the transfer directs the poorest individual. *L*_1,*c*_, *L*_1,*q*_ and *L*_1, *cq*_ do not change by the progressive transfer undirected to the poorest individual. Therefore, *L*_1,*c*_, *L*_1,*q*_, and *L*_1, *cq*_ satisfy the transfer principle when the transfer is directed toward the poorest individual. It is well known that the progressive transfer decreases the coefficient of variation *CV*_*z*_ and the Gini coefficient *G*_*z*_. However, *CV*_*z*_ and *G*_*z*_ have different signs in $L_{2,c}^{2}$ and $L_{2,q}^{2}$. The progressive transfer undirected to the poorest individual does not change $\mu_{z}$, decreases *CV*_*z*_, and *G*_*z*_, consequently, decreases $L_{2,q}^{2}$. But it increases *L*_1, *l*_ and $L_{2,c}^{2}$, and can increase $L_{2,cq}^{2}$ as the examples shown in Kim et al. [18]. A progressive transfer does not always guarantee a reduction in inequality.

**Fig 1:**
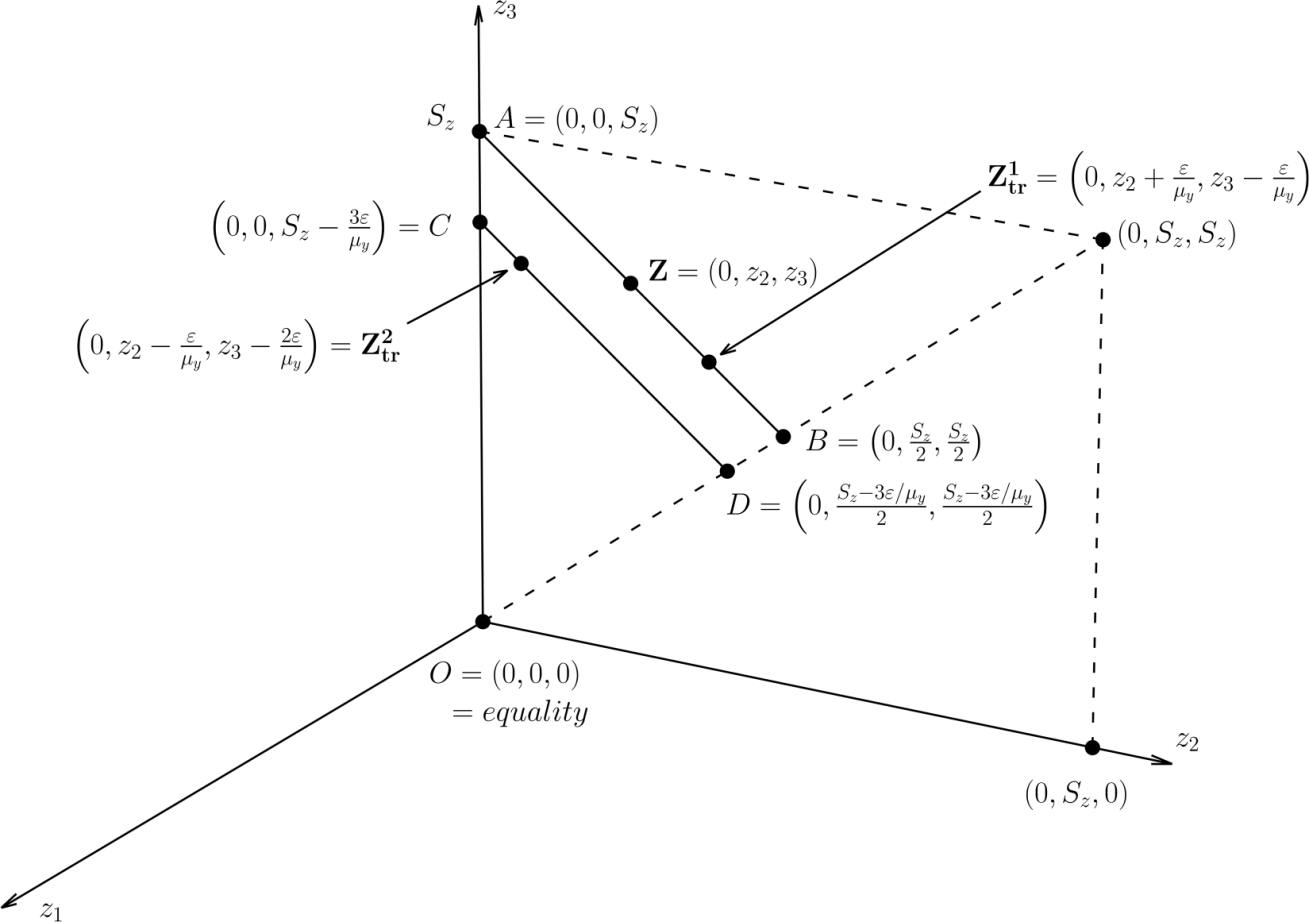
Effect of the progressive transfer on the departure from equality.

Kim et al. [18] visualized the effect of the progressive transfer by using CDQF of **z**. We illustrate it with a different graph. Suppose a population of 3 individuals. Its income distribution is $𝐲=\left(y_{1},y_{2},y_{3}\right)$, where $y_{1}\leq y_{2}\leq y_{3}$. The corresponding RUD income distribution is **z** on line **AB** in Fig 1. Remember that any RUD income distribution with *S*_*z*_ total RUD income lies on line **AB**. Consider a progressive transfer ϵ from *y*_3_ to *y*_2_ that preserves the mean *S*_*y*_. The RUD income distribution resulting from the transfer is ${𝐳}_{tr}^{1}$ on line **AB**. The RUD income distribution from any progressive transfer undirected to the poorest does not change the total RUD income and remains on line **AB**. Which of **z** and ${𝐳}_{tr}^{1}$ is closer to equality **O** depends on how to evaluate the departure from equality **O**. For example, the progressive transfer decreases $L_{2,q}^{2}$ but increases $L_{2,c}^{2}$ because the progressive transfer decreases *CV*_*z*_, and *G*_*z*_.

The RUD income distributions on line **AB** can be closer to equality **O** if the transfer directs to the poorest. Consider a progressive transfer ϵ from *y*_3_ to *y*_1_. The progressive transfer decreases the total RUD income by $3\epsilon /\mu_{y}$ and moves **z** on line **AB** to ${𝐳}_{tr}^{2}$ on line **CD**. All RUD income distributions with $\left(S_{z}-3\epsilon /\mu_{y}\right)$ total RUD income lies on line **CD**. It is obvious that line **CD** is closer to equality **O** than line **AB**. Similar explanation is possible for *n* ≥ 4 and needs (*n* − 1) dimensional hyperplanes corresponding to lines **AB** and **CD**. An efficient way to bring the RUD income distribution closer to equality is the progressive transfer to the poorest.

### Normalization

Normalization property requires that an inequality index has upper and lower bounds. The lower bound is the degree of inequality for perfect equality. The upper bound is the degree of inequality for perfect inequality, which is denoted by ${𝐲}_{pi}=\left(0,0,\ldots ,S_{y}\right)$. Perfect inequality is an income distribution in which the total income is the most unequally distributed. Cowell [2, p. 155] presents the upper bounds for the conventional indexes. A norm index D(𝐳) should satisfy the properties of the norm. Specifically, D(𝐳)≥0 and D(𝐳)=0 implies that **z** = 0. Since 𝐳=0 represents perfect equality, D(𝐳) takes zero lower bound for perfect equality.

The perfect inequality depends on the assumption that income is non-negative. The UD and RUD income-based approaches allow negative income and assume *S*_*y*_ > 0. Therefore, we can think of an income distribution more unequally distributed than **y**_*pi*_ and any given distribution. That implies that there is no perfect inequality and that D(𝐳) is not upper-bounded.

### Decomposability

The UD and RUD income-based approaches reveal that CDF and QF provide different information about inequality when the population is finite. We should note that populations under inequality evaluation are finite in practice. Table 1 presents a new type of decomposability, representation decomposability. Representation decomposability implies that CDF and QF deliver other information about inequality. We should integrate information from CDF and QF to assess the overall inequality. The overall inequality is the sum of CDF- and QF-based inequalities. Specifically, $L_{1,cq}=L_{1,c}+L_{1,q}$ and $L_{2,cq}^{2}=L_{2,c}^{2}+L_{2,q}^{2}$. *L*_1, *cq*_ and $L_{2,cq}^{2}$ are proper measures for the overall inequality. A simple stylized example helps explain why this representation matters. Consider two societies with the same subgroup means and the same within-group subgroup decomposition, but with different arrangements of income inside the subgroups. In one society, inequality is driven mainly by a small number of very low incomes, which changes the cumulative distribution more strongly. In the other, inequality is driven mainly by a small number of very high incomes, which changes the quantile structure more strongly. These two societies may look similar from the viewpoint of subgroup decomposability, because their subgroup summaries are the same, but they can differ meaningfully in CDF- and QF-based inequality. For this reason, representation decomposability adds information that subgroup decomposability alone does not capture, and it helps explain the analytical importance of the CDQF-based indexes. Next, we consider the usual subgroup decomposability. Suppose a population of *n* individuals decomposes into two subgroups with *n*_1_ and *n*_2_ individuals. Let **z**_1_ and **z**_2_ denote the corresponding decomposition of **z**. Then $\mu_{z,i}$, the mean of **z**_*i*_, is the *L*_1_ norm index of *i*-th subgroup. Since $\mu_{z}=\sum_{i=1}^{2}\alpha_{i}\mu_{z,i}$ where $\alpha_{i}=n_{i}/n$, *L*_1, *c*_, *L*_1, *q*_, and *L*_1, *cq*_ are the weighted sum of $\mu_{z,i}$s with weights $\alpha_{i}$s. We can further show that


$$L_{2,q}^{2}=\mu_{z}^{2}\left(1+CV_{z}^{2}\right)=\frac{\sum_{i=1}^{n}z_{i}^{2}}{n}=\sum_{i=1}^{2}\alpha_{i}L_{2,q,i}^{2},$$

where $L_{2,q,i}^{2}$ is the $L_{2,q}^{2}$ index of **z**_*i*_. Therefore, *L*_1, *c*_, *L*_1, *q*_, *L*_1, *cq*_, and $L_{2,q}^{2}$ have subgroup decomposability. However, *L*_1, *l*_, $L_{2,c}^{2}$, and $L_{2,cq}^{2}$ do not have subgroup decomposability.

## Simulation study

**Fig 2:**
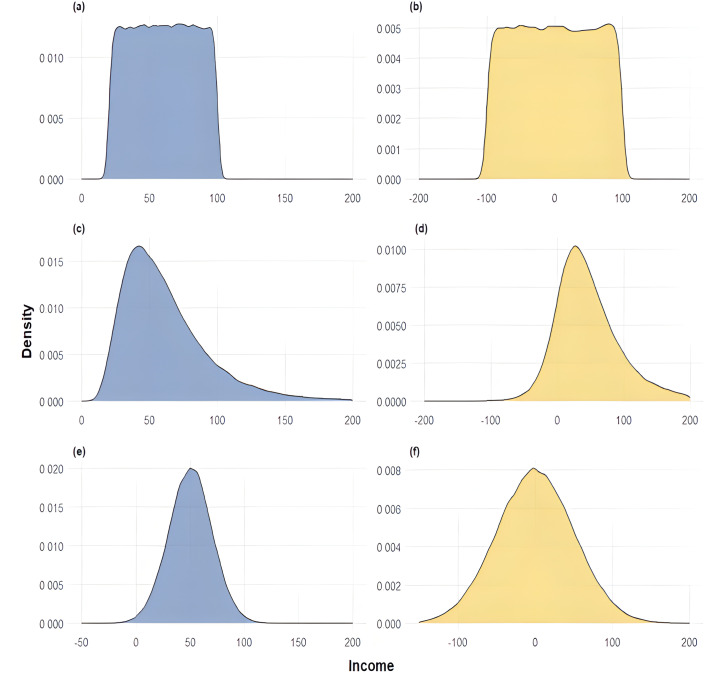
Density plots of simulated income using all six datasets.

A Monte Carlo simulation with 100 replications of 1,000 incomes each was conducted, and the generated data were then transformed into UD and RUD values. The six income-distribution datasets were generated to highlight different kinds of inequality: Dataset I was generated from a *Uniform*(20, 50) distribution, a rectangular, strictly positive density exhibiting mid, well-controlled inequality; Dataset II was generated from a *Uniform*(−100, 100) distribution, a systematic uniform density spanning negative and positive values, testing mixed-sign support; Dataset III was generated from a *Lognormal*(4, 0.5) distribution, wherein the underlying normal has mean *log*(4) and standard deviation 0.5, giving rise to a very right-skewed, heavy-tailed profile to test sensitivity to extreme upper-tail dispersion; Dataset IV was generated from a Johnson SU distribution with parameters γ=−1.1,δ=1.5, and *scale* = 50, producing mixed-sign skewness and a heavy tail; Dataset V was generated from a *Normal*(50, 20) distribution, a Gaussian law with mean 50 and standard deviation 20, yielding a symmetric strictly positive density and moderate mid-range inequality; and Dataset VI was generated from a *Normal*(0, 20) distribution, a centered Gaussian allowing both negative and positive values, hence testing behavior under standard mixed-sign support. For each RUD sample, we computed all the norm-based measures (mean, SD, CV, Gini, CDF- and quantile-based *L*_1_ and squared *L*_2_ norms, their combined CDQF versions, and the Lorenz-curve-based *L*_1_ norm) to compare performance across these distributional shapes. The distinguishing features of each dataset are summarized in Table 2. Fig 2 plots the density curves for these six datasets, showing how support and tail behavior influence each inequality measure.

**Table 2:** Income distribution datasets and inequality characteristics.

Dataset	Distribution	Inequality characteristics
I	*Uniform*(20,50)	Mild, well-controlled inequality
II	*Uniform*(−100,100)	Mixed-sign uniform support
III	*lognormal*(4,0.5)	Heavy-tailed upper tail inequality
IV	JohnsonSU(γ=−1.1,δ=1.5,scale=50)	Mixed-sign skewness
V	*Normal*(50,20)	Symmetric strictly positive, moderate inequality
VI	*Normal*(0,20)	Mixed-sign, moderate-range inequality

The full numerical results for all six datasets are presented in Table 3, while the income densities are displayed in Fig 2. All reported results are averaged over 100 simulation replicates for consistency. The mathematical expressions for the reported metrics can be found in Table 1.

**Table 3:** Mean values of inequality metrics (100 replicates) under six simulated datasets.

Dataset	$\mu_{z}\;$	*SD*_*z*_	*CV*_*z*_	*G*_*z*_	*L*_1,*c*_	*L*_1,*q*_	*L*_1,*cq*_	$L_{2,c}^{2}\;$	$L_{2,q}^{2}\;$	$L_{2,cq}^{2}$
I	0.666	0.384	0.577	0.333	0.666	0.666	1.331	0.222	0.444	0.590
II	−37.523	126.933	−0.613	−0.025	−37.523	−37.523	−75.046	−55.375	−110.749	−19.749
III	0.823	0.534	0.650	0.336	0.823	0.823	1.646	0.273	0.547	0.964
IV	3.012	1.131	0.389	0.200	3.012	3.012	6.025	1.216	3.432	4.648
V	1.296	0.398	0.311	0.175	1.296	1.296	2.593	0.536	1.072	1.608
VI	−173.057	451.811	−0.032	−0.018	−173.057	−173.057	−346.114	−213.847	−427.695	−641.542

Table 3 compares the response of the classical and norm-based inequality measures across six simulated datasets. Among the strictly positive distributions, Dataset I exhibits the lowest inequality ($\mu_{z}=0.66;G_{z}=0.33;L_{1,cq}=1.311;L_{2,cq}^{2}=1.034$). Dataset V produces a slightly higher dispersion ($\mu_{z}=1.296;G_{z}=0.175;L_{1,cq}=2.593;L_{2,cq}^{2}=2.993$), while Dataset IV remains between them ($\mu_{z}=0.823;G_{z}=0.336;L_{1,cq}=1.646;L_{2,cq}^{2}=1.511$). This ordering matches the familiar hierarchy of mild to moderate to strong right-tail inequality.

When income can be negative as well as positive, both the CV and Gini coefficient become unreliable (even negative or infinite) because they divide by a mean that is nearly zero. In our framework, we first shift every series so its minimum is zero and then compute all norms on these non-negative values. That simple UD transformation guarantees every inequality measure remains finite and interpretable, even when the raw data span both negative and positive values. For example, Datasets II and VI produce very large but well-defined *CDQF* norms, faithfully reflecting their extreme support width without any ad hoc adjustments.

Dataset VI delivers moderate skew metrics (*CV*_*z*_ = 0.389, *G*_*z*_ = 0.200) alongside large UD-based norms ($L_{1,cq}=6.025,L_{2,cq}^{2}=13.192$). The discrepancy between its CDF- and quantile-based components highlights how a handful of extreme positive outliers can dominate overall inequality, even though some incomes fall below zero. The *L*_1,*l*_ increases from 0.22 for the Dataset I to 1.216 for Dataset IV, accurately reflecting the growing curvature (inequality) of the Lorenz curve.

Within the axiomatic framework of this study, the *L*_1_-based norms stand out for their linear weighting of all deviations from perfect inequality. This linearity ensures stability: extreme outliers cannot disproportionately distort the index, and interpretability, because each unit of departure contributes equally to the total measure. By contrast, the squared *L*_2_ norm penalizes larger gaps quadratically, making it conceptually sensitive to heavy-tailed inequality but potentially less robust when a few extreme values dominate. In practice, this means *L*_1_ norms are preferable for analyses requiring resilience to outliers, whereas $L_{2}^{2}$ norms are more useful for highlighting distributions with pronounced tail disparities.

## Conclusion

The desirable properties of income inequality indexes are usually discussed in relation to income distributions. The conventional indexes have been examined to determine if they have the properties. Recently, the UD and RUD income-based approaches offered a different conceptualization of inequality and suggested the use of *L*_1_- and *L*_2_-norm indexes instead. In this paper, we proposed $L_{2}^{2}$, the square of the *L*_2_ norm, and investigated the properties of the *L*_1_- and $L_{2}^{2}$-norm indexes. We showed that the *L*_1_ and $L_{2}^{2}$ norm indexes satisfy the invariance properties and anonymity. We also showed that a progressive transfer can increase inequality and that a progressive transfer to the poorest individual improves inequality most effectively. The transfer principle is about the variation of income distribution. The progressive transfer decreases the variation of income distribution. However, it can increase the departure of income distribution from perfect equality. This result mainly affects the interpretation and measurement of inequality, and it may inform policy debates rather than directly prescribe policy changes. We further showed that inequality is not upper-bounded and that the norm indexes are not upper-bounded. We presented a new type of decomposability, representation decomposability, which differs from subgroup decomposability. Representation decomposability implies that CDF and QF provide different information about inequality and that the overall inequality requires integrating information from CDF and QF. The Lorenz curve is not considered for representation decomposability because the Lorenz curve derives from QF and provides equivalent information to QF as shown by *L*_1, *l*_ and $L_{2,q}^{2}$. *L*_1, *cq*_ and $L_{2,cq}^{2}$ have representation decomposability, while other norm indexes depend on one representation. In this sense, the *L*_1,*cq*_ and $L_{2,cq}^{2}$ indexes are appropriate measures of overall inequality. All the *L*_1_ norm indexes have subgroup decomposability except the *L*_1,*l*_.

This study is limited to theoretical properties and does not provide a large-scale empirical investigation of multidimensional inequality. Applying the proposed indexes to real-world data is an important direction for future research, and the framework may also be extended to cover multiple welfare dimensions.
